# Prospective cohort study on facial profile changes in infants with Robin sequence and healthy controls

**DOI:** 10.1007/s12519-024-00797-z

**Published:** 2024-04-05

**Authors:** Cornelia Wiechers, Julian Sowula, Karen Kreutzer, Christoph E. Schwarz, Christina Weismann, Michael Krimmel, Christian F. Poets, Bernd Koos

**Affiliations:** 1 Department of Neonatology, Tuebingen University Hospital, Calwerstrasse 7, 72076 Tuebingen, Germany; 2 Center for Cleft Lip, Palate and Craniofacial Malformations, Tuebingen University Hospital, Tuebingen, Germany; 3 Department of Orthodontics, Tuebingen University Hospital, Tuebingen, Germany; 4 Department of Oral and Maxillofacial Surgery, Tuebingen University Hospital, Tuebingen, Germany

**Keywords:** Infant, Mandibular catch-up growth, Pre-epiglottic Baton Plate, Robin sequence, Three-dimensional photography

## Abstract

**Background:**

Various conservative and surgical approaches exist to treat Robin sequence (RS), but their effects on facial profile and mandibular catch-up growth are unclear. A functional treatment concept, used in our centre for 25 years, includes an individualized palatal plate with a velo-pharyngeal extension and intensive feeding training.

**Methods:**

We performed a prospective study to objectively describe facial profiles in infants with RS treated with the above concept. Infants with isolated RS were admitted to our tertiary perinatal and national referral centre for craniofacial malformations between May 2018 and Nov 2019. Infants with RS received 3D-photographs during clinically indicated visits. Healthy controls were recruited from Dec 2018 to Sep 2019 and received 3D-photographs every 3 months. The digitally measured jaw index (JI), defined as alveolar overjet (O) x maxillary arch (U)/mandibular arch (L), and the soft tissue reference points A’-point, Nasion’, B’-point angle (ANB’), describing the relative position of maxilla to mandible, were evaluated. Linear mixed models were used to examine time trajectories in JI and ANB’**.**

**Results:**

A total of 207 3D images, obtained in 19 infants with RS and 32 controls, were analysed. JI and ANB’ decreased over time in both groups [for JI − 0.18 (95% CI − 0.25 to − 0.10); for ANB’: − 0.40° per month [(95% CI − 0.48 to − 0.32)]] but remained lower in controls [for JI − 2.5 (95% CI − 3.2 to − 1.8); for ANB’-1.7° (95% CI − 2.4 to − 1.0)]. Also, the ANB’ model showed a significant effect of the interaction term diagnosis x age.

**Conclusions:**

Based on longitudinal 3D images, we describe changes in objective parameters of facial profile in infants with and without RS during the first year of life. Our findings indicate catch-up growth in infants treated for RS.

Video Abstract

**Supplementary Information:**

The online version contains supplementary material available at 10.1007/s12519-024-00797-z.

## Background

Infants with Robin sequence (RS) suffer from respiratory and feeding difficulties, both related to their retro-positioned or underdeveloped mandible. Treatment protocols vary widely in RS, ranging from non-surgical (e.g., prone positioning, insertion of a nasopharyngeal tube, palatal plates or continuous positive airway pressure) to operative procedures (e.g., tongue-lip adhesion, mandibular distraction osteogenesis (MDO) or tracheostomy) [1–3]. However, the impact of these treatments on long-term mandibular growth has not been adequately studied. The literature is even inconclusive whether mandibular catch-up growth occurs at all in these children [1, 4–7].

Surgical procedures such as MDO attempt to improve respiration by gradually lengthening the mandible after an osteotomy using an internal or external distraction device as demonstrated by computed tomography (CT) scans [8–11]. According to our clinical experience, however, improved mandibular growth can also be achieved through a conservative approach developed at our centre that includes an individualized functional appliance with a velo-pharyngeal extension (Tuebingen Palatal or pre-epiglottic Baton Plate, TPP or PEBP), nutritional training and orofacial stimulation therapy. This was already demonstrated using manual determinations of the jaw index, which can quantify the extent of retrognathia in infants using a tape measure and micrometre depth gauge [12, 13]. Although determination of the jaw index is simple, cost-effective and non-invasive, it cannot be objectively verified. Since TPP treatment planning does not require radiologic imaging such as CT scans or magnetic resonance imaging (MRI), the latter techniques are not available to determine longitudinal measurements of mandibular growth or the facial profile [14]. Thus, to determine mandibular growth and facial profile changes, we set out to measure the jaw index digitally and longitudinally using 3D photography as a non-invasive, rapid and reproducible method and also the A’-point-Nasion’-B’-point (ANB’) soft-tissue angle, which is commonly used, in its bony correlate (ANB angle), in cephalometric analyses to measure the relative position of the maxilla to the mandible [12].

The aim of this study was to determine objective parameters as surrogates for mandibular growth and facial profile repeatedly throughout the first year of life in a prospective cohort of infants with isolated RS treated according to our protocol and healthy controls. We hypothesized that TPP treatment will result in mandibular catch-up growth in RS patients.

## Methods

### Participants

This prospective, longitudinal observational study was conducted at the Department of Neonatology, Department of Orthodontics and the Center for Cleft Lip, Palate and Craniofacial Malformations at Tuebingen University Hospital, Germany, a European Reference Network centre for rare craniofacial anomalies (ERN CRANIO).

Infants with isolated RS receiving treatment according to the Tuebingen protocol and admitted to our neonatal intermediate or intensive care unit were included in the study between May 15, 2018 and Nov 6, 2019 if they fulfilled inclusion criteria (singleton, gestational age at birth ≥ 37 0/7 weeks, no other congenital/facial anomalies or chromosomal disorders). Healthy term controls were recruited by the study team on the maternity ward between Dec 5, 2018 and Sep 27, 2019. Our study design did not allow for matching by age and sex.

Data were collected from electronic medical records and anthropometric parameters obtained during all study examinations. Calculation of Z-scores for weight, length and head circumference were based on normal values for age as reported by the WHO [15]; these parameters were computed using Perccalc® (Paedsoft, Tuebingen, Germany).

### Extraoral 3D photography and anthropometrical landmarks

In infants with RS, the first extraoral 3D photograph was taken immediately after admission to our center, i.e. before the onset of TPP treatment. Subsequent images were taken during routine follow-up visits, carried out approximately every 3 months. The control group examinations were also performed in 3-monthly intervals, i.e., at < 7 days (Visit 1), 3 (Visit 2), 6 (Visit 3), 9 (Visit 4) and 12 months (Visit 5).

For infants up to age three months, 3D images were performed in a separate room on our neonatal intermediate care unit in the supine position using a 3dMDhead system (3dMD Ltd, Brentford, London, UK) with six synchronised video cameras, with this set-up being developed specifically for this study. Subsequently, 3D images were taken in an upright position with infants sitting on their parent's lap in the department of orthodontics using a 3dMDhead system with nine synchronised video cameras. 3D data sets were analysed using Onyx Ceph software (Image Instruments, Chemnitz, Germany) and 3D Vultus (Software advice, INC, Austin Texas, USA), whereby the various anthropometric landmarks were entered manually and evaluated by two trained independent examiners blinded to group assignment. 3D images in which infants had their mouth open or both ears were not fully visible were excluded from analysis because the required distances could not be evaluated accurately.

**Fig. 1:**
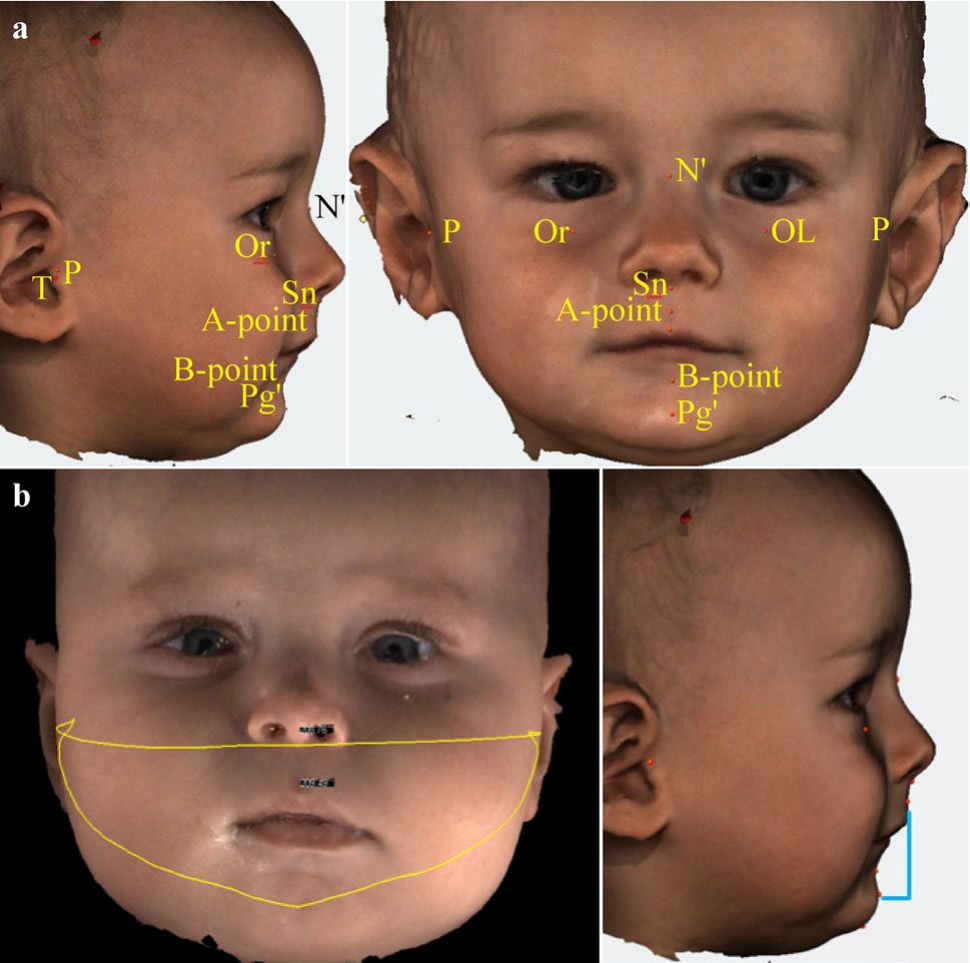
**a** Anthropometric landmarks and lines as used in the 3D images; **b** Digital jaw index (JI); **c** Digital A’-point nasion B’-point angle (ANB’) in a control infant (left) and an infant with RS (right). Consent for publications of the 3D-photographs of the two infants have been obtained from the parents

Anthropometric landmarks and lines used in the 3D images are listed in supplementary Table 1 and Fig. 1a. The JI is defined as alveolar overjet x maxillary arch/mandibular arch, measured in millimetres. It compares the size of the lower to that of the upper jaw (Fig. 1b) [12]. In the extraoral 3D photography, alveolar overjet, i.e. the maxillo-mandibular distance, was determined in the sagittal image from Pogonion’ (Pg’) to Subnasale (Sn). Maxillary arch was measured from right to left Tragion (T) through Sn, and mandibular arch from right to left T through Pg’. Higher values for this index are obtained with a smaller mandible and a larger alveolar overjet. We used soft tissue ANB’, which is a correlate to the bony ANB angle commonly used in cephalometric analyses, to determine the sagittal relationship between maxilla and mandible, i.e. between N` and the A’ and B’ points, respectively (Fig. 1c). Technical details of how measurements have been performed and their validity have been published as a medical thesis (see https://publikationen.uni-tuebingen.de/xmlui/handle/10900/145974).

### Tuebingen treatment protocol

An interdisciplinary team of neonatologists, paediatric sleep specialists, orthodontists, neonatal nurses, speech therapists and craniomaxillofacial surgeons are involved in our treatment protocol [16, 17]. Details are described in the supplemental material. Our study was registered with ‘Deutsches Register Klinischer Studien’ (trial number DRKS00017769), approved by the Ethics Committee of Tuebingen University Hospital and written informed parental consent obtained (reference number 738/2015 BO1) prior to study entry.

### Statistical analysis

Categorical variables were described using frequencies and percentages and continuous variables using medians and inter-quartile ranges (IQRs). Comparisons of *Z*-score values for weight between various time points were performed using the t-test. The time trajectory of the JI and ABN’ trend was modelled using a mixed effects model separately for each parameter. Diagnosis of RS (yes/no] was included in this model to compare potentially different trajectories of JI/ANB’ for the two groups. A linear mixed-effect model was implemented using intercept, postnatal age (time in months), and diagnosis as fixed effects. Subject (nested in diagnosis group) was included as random effect. Interaction variables of time-by-diagnosis were included as fixed effects. JI/ANB’ were the dependent variables. Significance was determined using 95% confidence intervals for fixed effects. The mixed model was implemented in JMP (version 16.0 Cary, NC, US), all other analyses using SPSS Statistics Version 27, IBM, Armonk, USA). For all comparisons, the level of significance was *P* < 0.05.

## Results

### Participants of the RS cohort

**Fig. 2:**
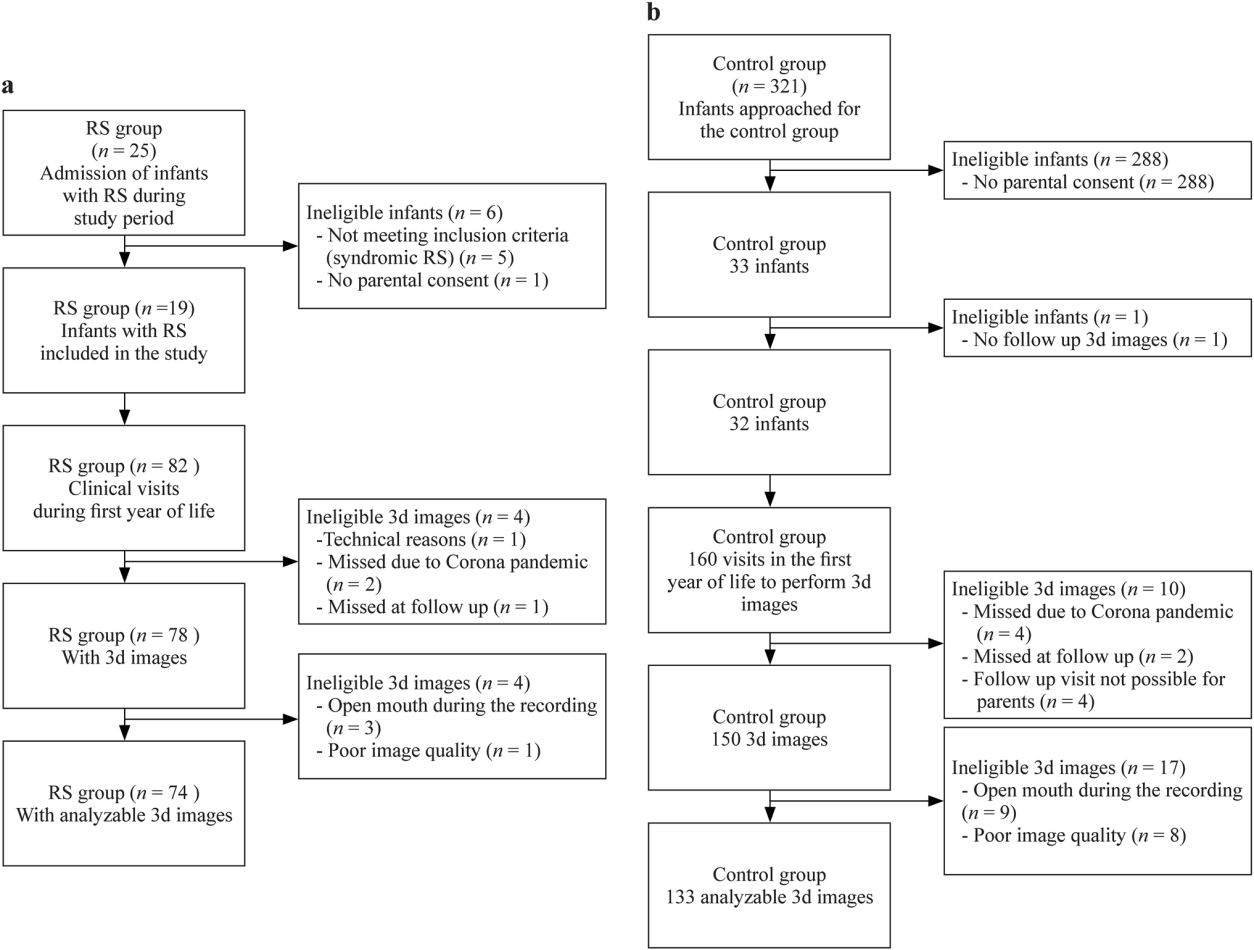
Flowchart of infants in the Robin sequence (RS; **a**) and control groups (**b**), follow-up visits and analyzable 3D images

During the recruitment period, a total of 20 infants with isolated RS were admitted to our centre, all families were approached and 19/20 (95%) agreed to participate, resulting in a final sample of 19 infants with RS (Fig. 2). All patients with RS presented with a cleft palate; only two had been suspected antenatally of having RS. One infant with RS was inborn; median age at admission to our centre was 19 (13–36) days and median duration of the initial hospital stay was 14 (13–29) days. The median obstructive apnoea index (OAI) decreased from 15.2 (7.0–30.0) events per hour at first admission to 1.5 (0.9–2.1; *P* < 0.001) events per hour at discharge after TPP fitting (Supplementary Fig. 1). All infants tolerated TPP treatment, but two required attaching a short tube to the pharyngeal extension; no further ventilatory support was required. One infant with RS was discharged on caffeine therapy due to apnoea of prematurity (gestational age 29 4/7 weeks, post discharge duration of therapy: 4 weeks). None of the other infants received any respiratory stimulant (e.g., caffeine) during hospitalisation or at discharge. Basic demographics are shown in Table 1.

**Table 1:** Clinical characteristics of patients and controls

Characteristics	RS patients	Control group
	(*n* = 19)	(*n* = 32)
Birth		
Sex (female)	9 (47%)	14 (47%)
Gestational age at birth (wk)	39.4 (38.1–40.0)	39.9 (38.9–40.4)
Birth weight (g)	3120 (2820–3580)	3380 (3130–3525)
Z-Score _Birth weight_	− 0.4 (− 0.9–0.4)	0.0 (− 0.3–0.4)
Head circumference (cm) _Birth_	34.5 (33.3–35.0)	35 (34.0–36.0)
First admission with TPP fitting		
Age at admission (d)	19 (13–37)	
Duration of hospital stay (d)	14 (13–29)	
Weight (g) Admission	3520 (3192–3859)	–
Discharge	3960 (3507–4439)	
Z-Score _weight_		–
Admission	− 0.57 (− 1.44 to − 0.33)	
Discharge	− 0.79 (− 1.34 to − 0.53)	
Obstructive apnoea index (OAI)		–
Before TPP fitting^a^	15.2 (6.8–30.0)	
After TPP fitting	1.4 (0.7–2.1)	
Received respiratory support (*n*)		–
Admission	5^a^	
Discharge	0	
Nasogastric tube (*n*)		–
Admission	10	
Discharge	3	
Duration of TPP use (d)	179 (131–211)	–
First 3d measurement		
Age (d)	27 (13–42)	2 (2–3)
Weight (g)	3530 (3140–3907)	3380 (3130–3538)
Digital jaw-index (mm)	19.3 (17.6–20.1)	12.3(10.0–14.2)
Maxillary arch (mm)	17.4 (16.5–18.3)	17.3 (16.8–17.9)
Mandibulary arch (mm)	16.5 (15.5–17.5)	16.7 (16.2–17.4)
ANB’	22.0 (18.0–25.7)	15.8 (13.1–17.0)
Last 3d measurement		
Age (d)	376 (299–416)	367 (355–376)
Weight (g)	9330 (8000–10,000)	9400 (8570–10,575)
Z-Score _Weight_	− 0.1 (− 0.9–0.6)	0.1 (− 0.4–1.1)
Digital Jaw-Index (mm)	15.8 (14.3–18.0)	10.7 (8.3–13.9)
Maxillary arch (mm)	20.8 (20.3–21.8)	21.3 (21.0–21.8)
Mandibulary arch (mm)	20.8 (19.9–22.1)	21.8 (21.2–22.2)
ANB’	15.0 (13.8–16.8)	12.3 (10.3–15.7)

^a^In one infant, a sleep study could not be performed due to the need for mechanical ventilation, and one sleep study had to be terminated early due to severe upper airway obstruction

Upon admission, 5 (26%) infants needed respiratory support: one (5%) invasive mechanical ventilation, 3 (16%) binasal continuous positive airway pressure (CPAP) and 1 (5%) CPAP via a pharyngeal tube

### Control group participants

A total of 321 parents of healthy, singleton term newborns were approached for the control group and 33 (10%) agreed to participate. In 32 of these infants (47% girls), 3D images could be obtained during the 1st year of life (Fig. 1). Demographics of the control group are shown in Table 1 and Supplementary Table 2.

### 3D photography

**Fig. 3:**
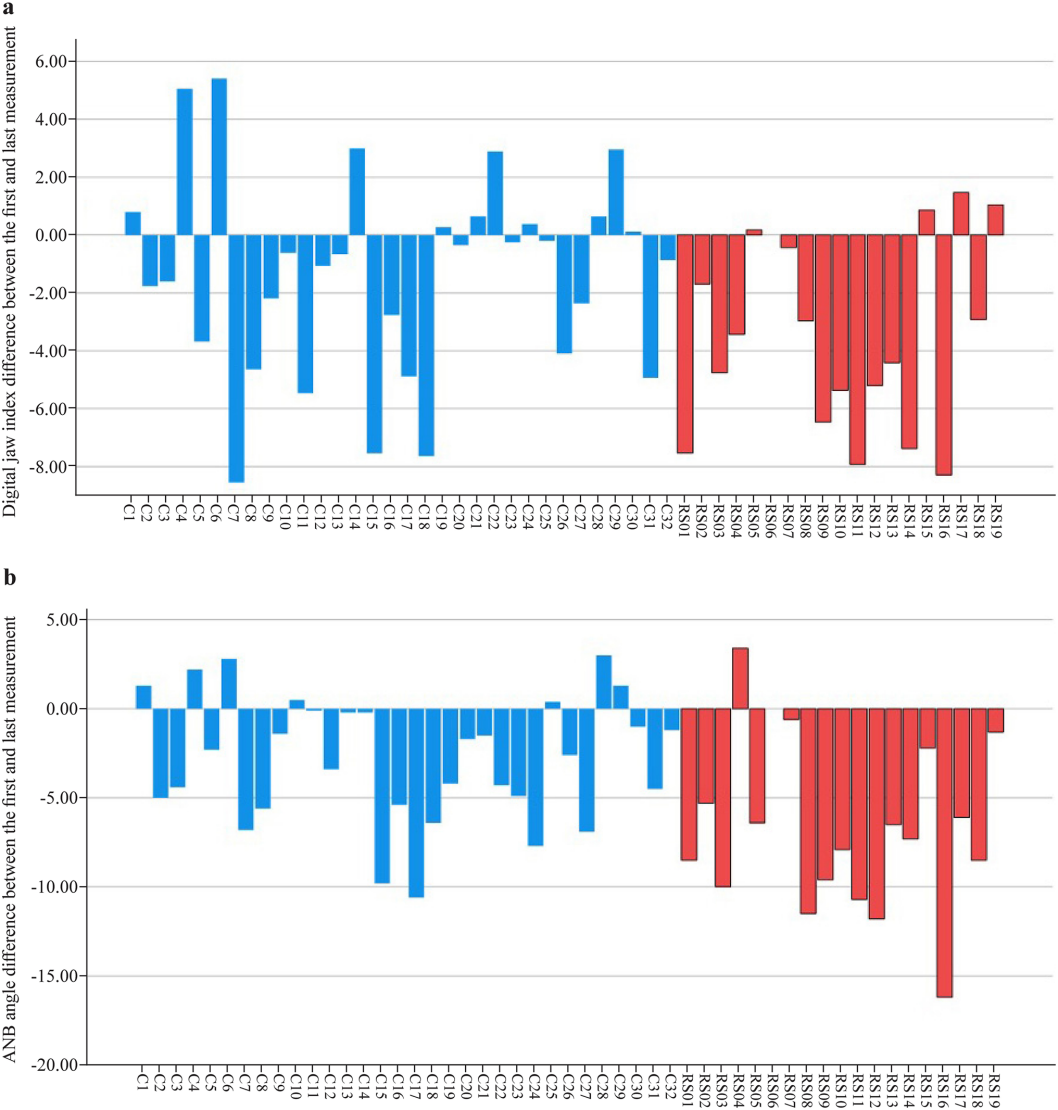
Difference between first and last measurement **a** Digital Jaw Index (JI); **b** A’-point Nasion’ B’-point angle (ANB’). Orange = RS group; blue = control group (**c**)

In both cohorts, a total of 228 images were taken using extraoral 3D photography; twenty-one 3D images (9%) were excluded for poor quality (i.e. open mouth during the recording or poor image quality). Thus, a total of 207 recordings could be evaluated (Fig. 1). Figure 3 shows difference between the first and last measurement of the Digital Jaw Index and ANB'. Interobserver agreement for these measurements was high (https://publikationen.uni-tuebingen.de/xmlui/handle/10900/145974, p73).

**Fig. 4:**
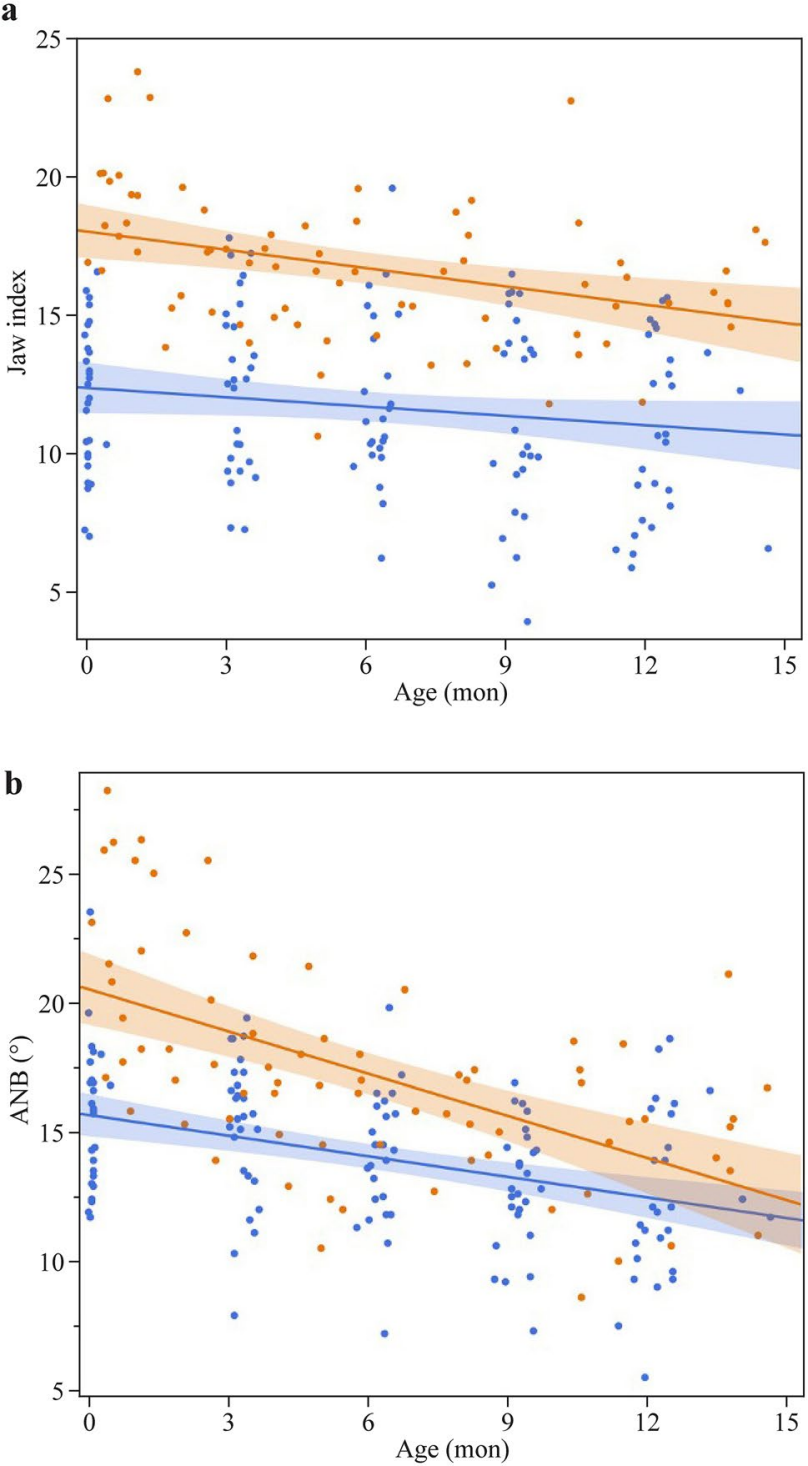
Linear mixed-effect model **a** Digital Jaw Index (JI); **b** A’-point Nasion’ B’-point angle (ANB’). Orange: RS group; blue: control group

In both, JI and ANB’ linear mixed effects models, there was a significant effect of RS diagnosis and time trajectory in the first year of life. Table 2 summarizes estimates (95% CI] of the linear effect models for the two parameters JI and ANB’. The models indicate that JI and ANB’ decreased over time in both, healthy infants and those with RS [for JI by − 0.18 (95% CI − 0.25 to − 0.10); for ANB’: − 0.40 (95% CI − 0.48 to − 0.32) degrees per month]. Furthermore, JI and ANB’ were lower in controls compared to RS [for JI: − 2.5 (95% CI − 3.2 to − 1.8); for ANB’: − 1.7 (95% CI − 2.4 to − 1.0) degrees]. For the ANB’, but not the JI model, the interaction term diagnosis x age was found to be statistically significant (Table 2, Fig. 4).

**Table 2:** Estimates (median, 95%CI) of the linear mixed-effect model for Jaw Index (JI) and A’-point nasion B’-point angle (ANB’). Each point represents one image from which measurements were taken

Variables	Model		
	Coefficient	(95% CI)	*P*-value
JI			
Intercept	15.2	(14.4 to 16.0)	< 0.001
Diagnosis (ref: RS)	− 2.5	(− 3.2 to − 1.8)	< 0.001
Age (mon]	− 0.18	(− 0.25to − 0.10))	< 0.001
Diagnosis x age (month-6.12)	0.07	(0.00 to 0.14)	0.054
ANB’			
Intercept	18.0	(17.2 to 18.9)	< 0.001
Diagnosis (ref: RS)	− 1.7	(− 2.4 to − 1.0)	< 0.001
Age (mon]	− 0.40	(− 0.48 to − 0.32)	< 0.001
Diagnosis x age (month-6.12]	0.11	(0.04 to 0.19)	0.002

*JI* Jaw Index, *ANB’* A’-point nasion B’-point angle, *RS* Robin sequence

## Discussion

This is the first prospective study to describe facial profile changes in infants with RS as well as in healthy controls using digital JI and ANB’. The aim of this study was to determine objective facial profile parameters as surrogates for mandibular growth throughout the first year of life. We hypothesized that there is catch-up growth of the mandible in infants with RS during TPP treatment.

Initially, both evaluated parameters showed a significant difference between infants with RS and healthy controls. Also, we found a decrease in digital JI and ANB’ during the first year of life in both groups. In both, JI and ANB’ linear mixed models, there was a significant effect of RS diagnosis, subsequent treatment and time trajectory. In addition, the ANB’ model showed a significant effect of the interaction term diagnosis x time. We interpret this finding as indicative of mandibular catch-up growth in infants with RS.

Longitudinal studies of mandibular growth in RS patients after conservative treatment are rare and usually included only small numbers of patients [5–7, 16–21]. Whereas most longitudinal studies found similar rates of sagittal mandibular growth compared to controls, two studies supported the concept of mandibular catch-up growth [18, 21].

Currently, there is no standard method for longitudinally monitoring mandibular retrognathia and micrognathia in RS. The studies mentioned above to assess mandibular growth used lateral cephalograms, but mostly at preschool and school age [7, 16, 20, 21]. However, due to radiation exposure, cephalograms or CT imaging are not suitable for close clinical monitoring. MRI examinations in infants with RS are very limited due to their need for sedation, which often leads to worsening of upper airway obstruction due to a decreased muscle tone, as well as high effort and cost. Therefore, some studies have used the manually measured JI as an inexpensive, quickly performed method for determining retrognathia in infants, quantifying the degree of retrognathia in infants using a tape measure and a micrometer depth gauge [12, 22]. In 1999, Vegter et al. used the JI for longitudinal measurements in seven infants with RS and healthy controls at birth and age 6 and 12 months [22]. Tongue-lip adhesion was applied to five of these RS patients. During the 1-year study period, the jaw index decreased from 4.2 (range, 0.0–9.5) to 1.5 (range, 0.94–3.1) in healthy controls and from 12.1 (range, 6.5–13.8) to 4.3 (range, 2.2–7.7) in RS patients [22]. A few years ago, we also measured the JI manually in infants with RS treated with TPP and found that median JI decreased from 8.8 (6.3–11.3) at admission to 2.1 (2.0 – 4.0) at the 3-month follow-up, again suggesting mandibular catch-up growth [13]. However, these manual measurements were neither objective nor could they be blinded, which is the reason why we now used 3D photography and measured the JI digitally and blinded to timing of measurement and diagnosis.

For the ANB’ model, the interaction term diagnosis x age was found to be statistically significant, indicating postnatal catch-up growth. Although the exact pathogenesis of RS remains unclear, postnatal development of the mandible is a complex intra- and extrauterine process influenced by various functional or genetic factors [1, 23, 24]. Subsequent mandibular growth induced by our treatment approach may be explained as follows: First, the individual fitting of the TPP pushes the base of the tongue forward, relocating the mandible ventrally in a neutral position and widening the hypopharynx, thereby creating a functional stimulus to the condyles of the temporomandibular joint that promotes mandibular growth in the primary cartilaginous growth centres according to generally accepted growth concepts [25], also confirmed by a systematic review [26]. Second, daily applied manual orofacial therapy, e.g., according to Castillo-Morales®, supports the neuronal control of the complex orofacial functions and interactions. Finally, emphasizing the importance of predominantly oral feeding, as preferred by our concept, may strengthen the orofacial musculature, thereby providing a growth stimulus to the surrounding bone [27, 28]. Indeed, myo-functional treatment has been demonstrated to improve obstructive sleep apnoea in children [29]. Therefore, the paradigm of "form follows function" might at least partially explain the mandibular catch-up growth after TPP treatment. The ANB’ seems to be the ideal parameter to monitor growth longitudinally, whereas the JI is potentially better suited to discriminate between RS and healthy controls.

Surgically, mandibular retrognathia and micrognathia can be treated using MDO, which gradually lengthens the mandible using a distraction device [11]. After MDO, both mandibular body length and the ramus height increased significantly [30–33]. Also, improved upper airway size and sleep study results were seen in CT scans and polysomnography, respectively [8–10, 34]. However, considering its high complication rate (up to 34%), and the lack of long-term data, we consider it encouraging that our conservative, functional approach can achieve similar mandibular growth together with a resolution of OSAS [11, 35].

### Limitations

Our study has limitations. First, extraoral 3D photography captures the facial profile including the soft tissues, but cannot provide detailed bony dimensions of the mandible. Unfortunately, for ethical reasons, we could not perform lateral cephalograms or CT scans. Therefore, the objective parameters used are surrogate parameters for mandibular growth, but not direct measures. However, both evaluated parameters showed significant group differences. Second, the timing of 3D imaging in the RS group showed a higher temporal dispersion than in the control group due to a wider age range at admission and thus a different timing of follow-up appointments. An ideal study design would have compared infants with TPP treatment to another conservative (e.g., prone position, continuous positive airway pressure or high flow nasal cannula) or surgical approach. However, this would not have been accepted by the team due to our positive experience with the TPP. Nonetheless, our 3D images and presented data will allow for a comparison with other therapy approaches in the future.

Strengths of our study include the non-invasive, simple, standardized, objective and fast technique used for assessing the facial profile in infants with isolated RS. Furthermore, all infants with isolated RS could be prospectively studied in the first year of life, and all received the same treatment. In addition, we were able to determine longitudinal reference values for the facial profile using digital JI and ANB’ in healthy, Caucasian infants during the first year of life.

In conclusion, using longitudinal 3D images, we were able to show that in both, infants with RS treated with the Tuebingen therapy concept and healthy controls, the JI and ANB’ decreased during the first year of life. JI and ANB’ showed a significant influence of RS diagnosis during TPP and functional treatment on both mandibular parameters. Using the ANB’, we were able to show that infants with RS have mandibular catch-up growth during the first year of life. In the future, these data could be used to monitor treatment effectiveness as well as to compare the effects of different conservative and surgical therapeutic approaches on mandibular growth.

### Supplementary Information

Below is the link to the electronic supplementary material.

Supplementary file1 (DOCX 1707 KB)

## Data Availability

Data from this study are available upon reasonable request.
