# Supplementary material for: Prospective cohort study on facial profile changes in infants with Robin sequence and healthy controls
Source: World J Pediatr. 2024 Apr 5;20(6):581–9. doi: 10.1007/s12519-024-00797-z (PMC11239728; doi:10.1007/s12519-024-00797-z)
Supplement: Supplementary file 2 — Supplementary file1 (DOCX 1707 KB) [file 12519_2024_797_MOESM1_ESM.docx]

**Supplement**

*Tuebingen treatment protocol*

An interdisciplinary team of neonatologists, paediatric sleep specialists, neonatal nurses, speech therapists, orthodontists and craniomaxillofacial surgeons is involved in the treatment protocol, already described in detail elsewhere ^1,2^. In summary, the severity of upper airway obstruction (UAO) is assessed by a bedside baseline multichannel, computerized cardiorespiratory sleep study (Embla N 7000, MedCare, Reykjavik, Iceland) in supine position, usually during the second night after admission. The Tuebingen Palatal Plate (TPP) is an individual functional orthodontic appliance consisting of a velophayrngeal extension, a palatal base plate and two extraoral fixation bows. The velopharyngeal extension ends just above the epiglottis to push the tongue forward, thereby opening the upper airway. The indication for initiating TPP treatment is an obstructive apnoea index (OAI) >3 ^2-4^. Central, mixed and obstructive apnoeas are identified, and an OAI is calculated as the sum of all obstructive and mixed apnoeas per hour of total sleep time according to the criteria of the American Academy of Sleep Medicine (AASM) ^5,6^.

In our study, no sleep study could be performed in one infant due to mechanical ventilation, another sleep study had to be terminated due to severe UAO. The one child with mechanical ventilation who did not have a sleep study because her UAO was so severe was arbitrarily (and conservatively) assigned an OAI of 30 for statistical analysis of her sleep study results ^7^.

After intraoral 3D scanning of the maxilla using a Trios 3® intraoral scanner (3Shape, Copenhagen, Denmark)^8^, the TPP is designed. With an awake bedside fibreoptic nasopharyngoscopy using C-MAC® (Monitor 8403 ZX, Karl Storz, Tuttlingen, Germany) the correct position of the velopharyngeal extension is confirmed. The tip of this extension should end just above the epiglottis and the airway should appear completely open. The TPP is supplemented by early specialized oral feeding techniques, starting with an infant feeder (Finger Feeder, Medela, Baar, Switzerland) and followed by a special feeding bottle with variable milk flow (Playtex Drop-Ins® Playtex Products, Edgewell, North Bergen, NY) and stimulation of the oral musculature based on the Castillo-Morales® approach ^9,10^.

The TPP is worn continuously, with its fit and the mucosa regularly checked for the occurrence of pressure marks by the clinical team. It is usually removed briefly for cleaning and this check-up once daily. If pressure marks occur, the base plate is adjusted to release the according gingiva. After several days of treatment with a clinically well-fitting TPP, its effectiveness is confirmed by a second sleep study, with the aim of achieving an obstructive apnoea index OAI <3. The angle between the base of the plate and its velopharyngeal extension needs to be optimized if the OAI is still >3. Family education and involvement in handling the plate and in feeding techniques is started as early as possible. Infants are discharged home if they have (1) a well-fitting TTP without relevant pressure marks for 2-3 days, (2) an OAI<3 in the last sleep study and (3) show adequate weight gain.

After discharge, regular check-up in the department of orthodontics are performed to ensure the correct fitting and functioning of the appliance. In addition, infants are re-admitted for regular sleep studies including clinical and orthodontic follow-up in 3-monthly intervals throughout their first year of life. Approximately 3 months after discharge, infants require a larger TPP due to craniofacial growth, which is adapted during a 3-5 day inpatient stay. TPP treatment is usually discontinued at 6-8 months of age when the facial profile has harmonized, and the results of the sleep study show an OAI<1 after the TPP has not been worn for 10 days. Surgical closure of the cleft palate is done at around 12 months of age.


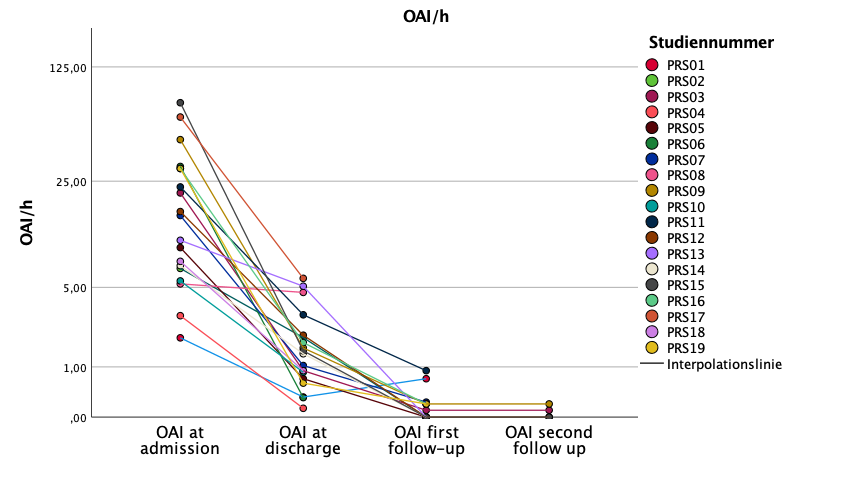


**Supplementary Figure 1** Obstructive apnoea index (OAI) in Robin sequence infants during first year of life

**Supplement table 1**

| Anthropometric landmarks | Landmark Symbol | Definition |
| --- | --- | --- |
| Orbitale | Or | The lower edge of the orbita |
| Porion | P | Most superior point of outline of external auditory meatus |
| Labialis superior | Ls | Most prominent protrusion of the upper lip |
| Subnasale | Sn | Midpoint of the angle at the columella point base the junction of the nose to the upper lip |
| Pogonion | Pg’ | Most anterior midpoint of the chin |
| Tragion | T | Notch above the tragus of the ear where the upper edge of the cartilage disappears into the skin of the face |
| Soft tissue Nasion’ | N’ | Nasofrontal groove |
| Soft tissue A’ point | A’ | Most concave point of anterior upper lip/sulcus labialis superior |
| Soft tissue B’ point | B’ | Most concave point in the concavity of the lower lip/ sulcus labialis inferior |
| Frankfurt horizontal plane (Tragus orbita line) |  | The line from the lower edge of the orbita to the porion; served as the reference point of the three-dimensional coordinate system ^11^ |

**Supplementary Table 2** Characteristics of healthy control group at 3D measurements throughout the first year of life

| **Characteristic** | **Visit 1** | **Visit 2** | **Visit 3** | **Visit 4** | **Visit 5** |
| --- | --- | --- | --- | --- | --- |
| Number of participants | 32 (100%) | 32 (100%) | 28 (87.5%) | 28 (87.5%) | 30 (90.6%) |
| **Infant characteristics** | | | | | |
| Age at measurement (days) | 2  (2-3) | 100  (94-103) | 190  (185 -193) | 278  (273-283) | 367  (359-373) |
| Weight (g) | 3225  (397) | 6135  (734) | 7920  (993) | 8928  (1041) | 9673  (1137) |
| Z score weight | -0.04  (-0.3-0.4) | -0.06  (-0.8-0.6) | 0.40  (-0.6-1) | 0.16  (-0.2-1.1) | 0.13  (-0.4-1.1) |
| Head circumference (cm) | 35 (1.2) | 40 (1) | 43 (1) | 45 (1.2) | 46 (1) |
| Body length (cm) | 51 (2.5) | 62 (2.9) | 69 (2.6) | 73 (2.9) | 76 (4) |
| **3D Images** |  |  |  |  |  |
| Analysable images (%) | 28 (84%) | 28 (88%) | 24 (86%) | 26 (93%) | 27 (90%) |
| Jaw-Index (mm) | 12.3  (10.3-13.9) | 12.6  (9.8-15.0) | 11.2  (10.1-14.8) | 10.6  (9.0-14.3) | 10.7  (7.7-13.7) |
| Maxillary arch (mm) | 17.3  (16.8-17.9) | 19.8  (19.3-20.3) | 21  (19.9-21.2) | 21.1  (20.8-21.5) | 21.4  (21.0-21.8) |
| Mandibulary arch (mm) | 16.7(  16.2-17.4) | 19.8  (19.1-20.8) | 21.2  (20.3-21.8) | 21.7  (20.8-22.2) | 21.8  (21.3-22.2) |
| ANB’ | 15.8  (13.2-16.9) | 15.6  (13.4-16.9) | 14.1  (12,0-16.0) | 12.8  (11.6-14.5) | 11.9  (10.1-15.7) |

Abbreviation: ANB’= A’-point Nasion’ B’-point angle

Values are given as median (IQR) or n (%). 3D Measurements were performed from birth to 12 months of age in 3-monthly intervals, i.e., at <7 days (Visit 1), 3 months (Visit 2), 6 months (Visit 3), 9 months (Visit 4) and 12 months (Visit 5).

1. Wiechers C, Arand J, Koos B, Poets CF. Evidence and practical aspects of treatment with the Tübingen palatal plate. *Semin Fetal Neonatal Med*. Dec 2021;26(6):101281. doi:10.1016/j.siny.2021.101281

2. Poets CF, Koos B, Reinert S, Wiechers C. The Tubingen palatal plate approach to Robin sequence: Summary of current evidence. *J Craniomaxillofac Surg*. Aug 20 2019;doi:10.1016/j.jcms.2019.08.002

3. Buchenau W, Urschitz MS, Sautermeister J, et al. A randomized clinical trial of a new orthodontic appliance to improve upper airway obstruction in infants with Pierre Robin sequence. *J Pediatr*. Aug 2007;151(2):145-9. doi:10.1016/j.jpeds.2007.02.063

4. Poets CF, Wiechers C, Koos B, Muzaffar AR, Gozal D. Pierre Robin and Breathing: What To Do and When? *Pediatr Pulmonol*. Feb 13 2021;doi:10.1002/ppul.25317

5. Berry RB, Gamaldo CE, Harding SM, et al. AASM Scoring Manual Version 2.2 Updates: New Chapters for Scoring Infant Sleep Staging and Home Sleep Apnea Testing. *J Clin Sleep Med*. Nov 15 2015;11(11):1253-4. doi:10.5664/jcsm.5176

6. Standards and indications for cardiopulmonary sleep studies in children. American Thoracic Society. *Am J Respir Crit Care Med*. Feb 1996;153(2):866-78. doi:10.1164/ajrccm.153.2.8564147

7. Muller-Hagedorn S, Wiechers C, Arand J, et al. Less invasive treatment of sleep-disordered breathing in children with syndromic craniosynostosis. *Orphanet J Rare Dis*. Apr 23 2018;13(1):63. doi:10.1186/s13023-018-0808-4

8. Weise C, Frank K, Wiechers C, et al. Intraoral scanning of neonates and infants with craniofacial disorders: feasibility, scanning duration, and clinical experience. *Eur J Orthod*. May 24 2022;44(3):279-286. doi:10.1093/ejo/cjab068

9. Limbrock GJ, Castillo-Morales R, Hoyer H, Stover B, Onufer CN. The Castillo-Morales approach to orofacial pathology in Down syndrome. *Int J Orofacial Myology*. Nov 1993;19:30-7.

10. von Lukowicz M, Herzog N, Ruthardt S, Quante M, Iven G, Poets CF. Effect of a 1-week intense myofunctional training on obstructive sleep apnoea in children with Down syndrome. *Arch Dis Child*. Mar 2019;104(3):275-279. doi:10.1136/archdischild-2018-315064

11. Freimann FB, Luhdo ML, Rohde V, Vajkoczy P, Wolf S, Sprung C. The Frankfurt horizontal plane as a reference for the implantation of gravitational units: a series of 376 adult patients. *Acta Neurochir (Wien)*. Jul 2014;156(7):1351-6. doi:10.1007/s00701-014-2076-y
